# A mobile minimally invasive interventional shelter: a new answer to on-spot emergency treatment of large arterial injuries?

**DOI:** 10.1186/s13049-015-0144-9

**Published:** 2015-09-04

**Authors:** Ming Liang, Jingjing Rong, Jingyang Sun, Tianming Yao, Fengqi Xuan, Lijun Zhao, Fei Li, Xiaozeng Wang, Yaling Han

**Affiliations:** Department of Cardiology, General Hospital of Shenyang Military Region, Shenyang, 110016 China

## Abstract

**Background:**

Severely destructive disasters can often lead to heavy casualties. Large arterial injury in disasters, particularly, often results in high mortality and morbidity. Although minimally invasive intervention has achieved positive effects in diagnosing and treating vascular injuries, it is still unavailable at the disaster area of any country due to lack of on-spot catheterization labs. This study aimed to test the feasibility of adopting interventional techniques to treat haemorrhage of large arterial injuries in remote and austere wild environments after severely destructive disasters, by using a new mobile intervention suite we developed—the mobile minimally invasive interventional shelter (MIS).

**Methods:**

Large animal models of aortic and femoral arterial injuries were established using a newly developed medium vehicle-mounted digital subtraction angiography (DSA) machine in MIS. The endovascular stent-graft exclusion and balloon occlusion combined with surgical hemostasis were performed respectively following the protocols for rapid interventional therapy. The treatment capacity of the shelter was evaluated based on its stability, surgery duration and the clinical results.

**Results and discussion:**

The stability of the medical devices in MIS directly relates to the efficiency and success rate of interventional treatment. The newly developed vehicle-mounted DSA machine showed good imaging performance and the operation of all equipments and devices in MIS were stable in interventional procedures. All the interventional treatments for large arterial injuries were performed smoothly. The average time for treating abdominal aortic injury and femoral arterial injury was 23 ± 11 and 55 ± 17 min, respectively. And the operation success rate reached 100 %.

**Conclusion:**

It is feasible to perform interventional operations to control haemorrhage of large arterial injuries in MIS outside hospital. The MIS has a great potential to save patients from dying of hemorrhagic shock due to lack of effective treatment devices and approaches in remote and austere wild environments, such as in disaster areas.

## Background

Natural disasters often lead to heavy casualties. Among the injuries inflicted by natural disasters, severe trauma to large arteries and hemorrhagic shock are the most common causes of death and disability in the injured persons. Hence, it is essential to provide timely and effective pre-hospital emergency care which can significantly reduce the mortality and incidence of disability [1]. However, although minimally invasive interventional diagnosis and treatment of traumatic vascular injuries has achieved positive effects [2–4], it is rarely performed in on-spot and pre-hospital emergency care due to a shortage of mobile interventional equipment as well as power supply limitations. So far, on-spot interventional therapy in disaster assistance has been scarcely reported [5, 6]. Thus, we proposed and designed a new vehicle-mounted medical equipment system—the mobile minimally invasive interventional shelter (MIS) to provide on-spot medical assistance for emergency intervention in disaster areas [7–9]. The present study evaluates the potential of on-spot emergency interventional diagnosis and treatment of injuries to large arteries using MIS by large animal experiments in the simulated disaster environment.

## Methods

### Introduction to MIS

**Fig. 1:**
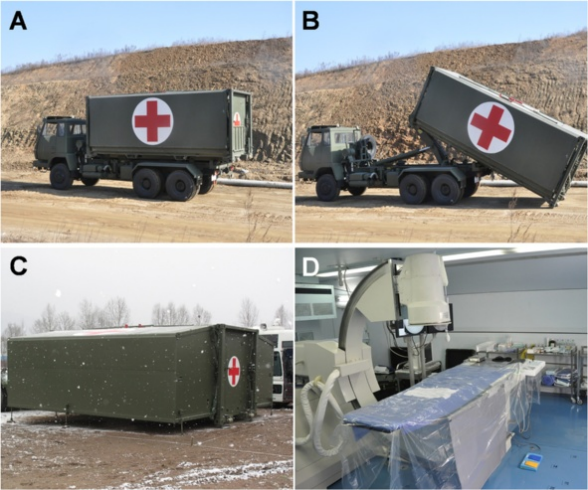
The vehicle-mounted state, self-unloading, unfolding and inner facilities of MIS. **a**. the appearance of unfolded MIS; **b**. the MIS was unloading; **c**. the unfolded MIS on flat ground; **d**. the internal facilities of MIS in working state

The MIS was designed by our research group and built by our cooperation unit (Beijing Smart medical equipment co., LTD). 19.8 tons in weight, MIS can be carried by truck, transport plane, railway or cargo ship to the targeted area (Fig. 1a and b). The shelter of MIS is folding and is extensible bilaterally in working state (Fig. 1c and d). It can be set up on flat ground, covering an area of 40 ~ 50 m^2^. The dimension of the shelter when folded and extended is 6.05 m × 2.44 m × 2.44 m and 6.05 m × 6.26 m × 2.44 m, respectively. The time of unfolding the shelter, and performing disinfection and preoperative preparation was 33 ± 5 min [7]. The whole equipment system of MIS comprised diesel generator power lighting, air purification, heating, oxygen and water supply, and some other auxiliary systems. Several necessary minimally invasive interventional apparatus and operating instruments sets were installed into the shelter, including a newly developed medium vehicle-mounted interventional angiography system, operating tables, defibrillator, invasive blood pressure monitor, high pressure injector, x-ray protective clothing, and an image acquisition system. In addition, the MIS was equipped with specialized operating instruments sets for emergency surgery.

The X-ray system of MIS is a novel medium interventional angiography system developed by our research group (power 16 KW, maximum pulse speed 12 FPS). The X-ray radiation dosages in MIS were 39.55uGy/s, 247.4uGy/h, 90.3uGy/h and 39.4uGy/h, corresponding to 0, 1, 2 and 3 m far away from the tube central of C-arm, respectively, and it is safe to carry out interventional diagnosis and treatments in MIS [9]. Besides meeting the clinical needs, the design, manufacture and assembling of all parts of the X-ray machine also met the stringent standards of the vehicle-mounted equipment as well as other equipments in MIS, including adapting to the high or low temperature environment ( −41–46 °C), resistance to shock, moisture and dust, and anticorrosion. In addition, for adapting to the austere wild conditions and achieving sterilization for interventional operation, sterilizing equipment were installed and antibacterial materials were used in MIS, including laminar flow equipment and filter devices, ultraviolet disinfection equipment, as well as antimicrobial films and metallic materials. The number of colonies in the sterilized shelter body was 88 ± 18 cfu/m^3^ [7], which met the clean-level requirements of simple surgery. Therefore, the MIS is a novel and safe medical equipment system, not a miniature of hospital catheter lab.

### Experimental animals

Eleven adult beagle dogs (male, 21–25 kg) were used in this research. Six of them were used to establish abdominal aortic injury models and the other five to establish femoral arterial injury models. The animals were provided by the Experimental Animal Center of Shenyang Northern Hospital and the experiment was supervised and authorized by the Ethics Committee of Shenyang Northern Hospital. All the dogs received humane care in accordance with the guidelines published by the National Society for Medical Research (Principles of Laboratory Animal Care) and the National Institutes of Health (Guide for the Care and Use of Laboratory Animals).

### Modeling large arterial injury

The MIS was unfolded at different times and different locations in the field. The dogs were then fixed on the operating table and anesthesia was induced by intravenous injection of propofol (3 mg/kg). Endotracheal intubation was performed and the anesthesia was maintained at 10 mg/kg/h. The blood pressure and heart rate of the dogs were monitored closely during the experiment. Following skin preparation, disinfection and local anesthesia, an arterial sheath was inserted into the femoral artery by Seldinger technique. And then, 5000U of heparin was injected from the side tube and 1000 U/h was added as supplement.

#### Aortic injury model

**Fig. 2:**
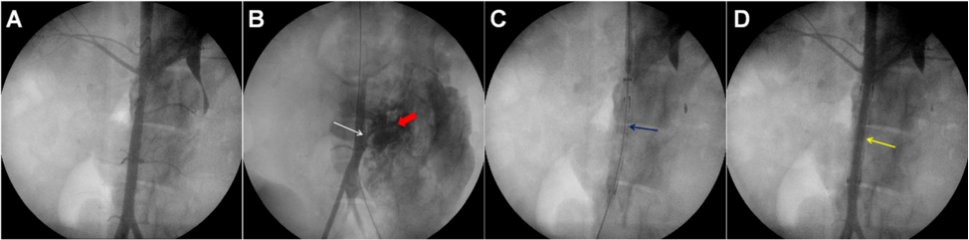
The angiography of normal abdominal aorta, aortic injury model and hemostasis after endovascular stent-graft exclusions. **a**. angiographic image of normal abdominal aorta; **b**. aortic injury model (*white arrow* shows the injury site and *red arrow* shows the retention of contrast agent); **c**. stent graft at the landing zone where the lesion was covered; **d**. successful endovascular stent-graft exclusions and hemostasis (*yellow arrow* shows the stent graft and hemorrhage is under control)

Using loach guide wire, an angiography catheter was inserted along the sheath into the left femoral artery to perform abdominal aortic angiography (Fig. 2a). After removing the catheter, a Swartz sheath was inserted, with the tip positioned against the predetermined site in the abdominal aortic wall between the origins of renal and iliac arteries. Then, the Swartz sheath was pushed forward repeatedly to damage the aorta for establishing the injury model. Angiography was performed to confirm the injury based on the obvious leakage of contrast agent from the aorta (Fig. 2b). Later, the sheath and catheter were drawn out and the puncture site was dressed with appropriate pressure.

#### Femoral artery injury model

**Fig. 3:**
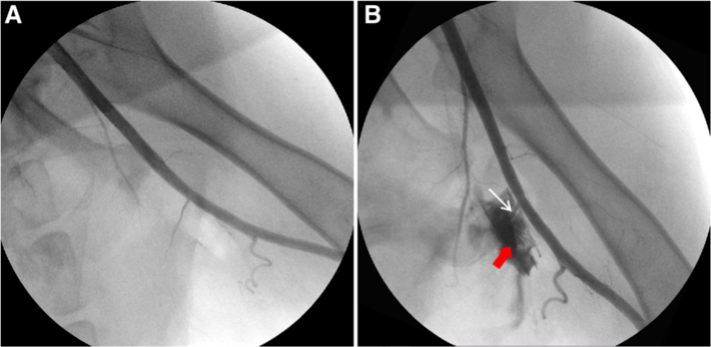
The angiography of normal femoral artery and arterial injury model. **a**. angiographic image of normal femoral artery; **b**. arterial injury model (*white arrow* shows the injury site and *red arrow* shows the retention of contrast agent)

6F arterial sheath and angiography catheter were inserted into the right femoral artery successively. After performing angiography at the origin of left femoral artery, a large branch was chosen to establish the injury model (Fig. 3a). Then a Swartz sheath was inserted into the predetermined branch and pushed forward repeatedly to damage the branch of femoral artery. The leakage from the lesion and retention of contrast agent were observed clearly under DSA to confirm the hemorrhage (Fig. 3b).

### Interventional treatments

#### Endovascular stent-graft exclusions for aortic injury

After creating the abdominal aortic injury model, a new 6F arterial sheath was inserted into the right femoral artery. And angiographic intervention was performed again to confirm and locate the lesion of the injured aorta. Next, a guiding catheter was pushed forward along the guide wire to the landing zone and the stent graft (HT1616-080-0000, MicroPort, Shanghai) was located at the lesion site to cover the whole lesion (Fig. 2c). Then, balloon dilation was performed with nominal pressure to release the stent graft. After that, angiography was performed repeatedly to guide how to appose the stent well and to observe the hemostatic effect (Fig. 2d). Right after operation, the catheter was withdrawn and the puncture site was dressed with appropriate pressure. The operation time was recorded. Anti-shock treatment and intravenous fluid replacements were performed during the operation. Finally, the dogs were sent back to the Experimental Animal Center.

#### Balloon occlusion combined with surgical hemostasis for femoral artery injury

**Fig. 4:**
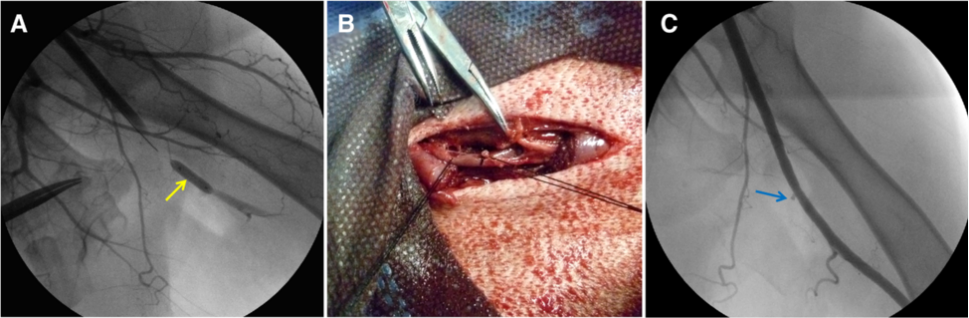
Temporary balloon occlusion combined with surgical hemostasis. **a**. successful balloon occlusion of the injured femoral artery (*yellow arrow* shows the balloon and hemorrhage is under control); **b**. The injured branch of femoral artery was ligated after temporary balloon occlusion; **c**. angiographic image after surgical hemostasis and balloon withdrawal (*blue arrow* shows the stump of injured femoral arterial branch after ligation)

Angiography was performed to verify the lesion and hemorrhage of the injured branch of right femoral artery. Then, a balloon catheter (EMPIRA NC 3.5 mm × 10 mm, Johnson &Johnson Cordis, USA) was inducted by the guiding wire to the intended releasing site, which was proximal to the arterial lesion. Air of 4–5 atmospheres was injected to inflate the balloon until the blood flow in the injured artery was occluded completely (Fig. 4a). Afterwards, the surgical exploration and hemostasis were performed using operating instruments sets which were equipped in MIS as essential equipment. The body surface projection of the lesion was determined firstly according to the angiographic image. After local anesthesia, the skin was incised and subcutaneous tissue was then detached layer by layer. Afterwards, the hematoma was removed and the lesion was exposed. 2–0 sutures were used to repair or ligature the lesion (Fig. 4b). Later, the balloon was withdrawn and the effect of balloon occlusion combined with surgical hemostasis was demonstrated by angiography (Fig. 4c). Finally, the incision was closed, and the puncture site was dressed with pressure after catheter was removed.

#### Statistical analysis

The continuous data were described as mean ± standard deviation. Data were analyzed using SPSS 12.0 and a *p* value <0.05 was considered statistically significant.

## Results

Angiographic results which showed qualified images with clear blood vessels and good contrast ratio were obtained in the MIS. The newly developed medium vehicle-mounted interventional angiography system, as well as other medical equipment in the MIS, was stable with no malfunction during operation. All the aortic and femoral arterial injury models were established successfully.

### Endovascular stent-graft exclusions

The urgent interventional hemostasis using endovascular stent-graft exclusion for abdominal aortic injury and hemorrhage was performed successfully in the six dog models in MIS. Slight contrast agent extravasation was found in one dog by angiography immediately after primary endovascular stent-graft exclusion. Afterwards, another stent graft was released as remedy and then the extravasation disappeared. The technical success rate of endovascular stent-graft exclusion in MIS was 100 % and the mean operation time was 23 ± 11 min. There were not significant differences in mean blood pressure between different stages during the experiment, including before model establishment, 1–2 min after model establishment and 2 h after operation (*p* >0.05, Table 1). However, the heart rate after model establishment was obviously higher than that before model establishment (*p* <0.05, Table 1)

**Table 1:** Hemodynamics of Endovascular stent-graft exclusion experiment (n=5)

Hemodynamic parameters	Before modeling establishment	After modeling establishment	Post-operation
SBP(mmHg)	178.4±13.6	181.5±19.7	183.5±17.3
DBP(mmHg)	131.2±12.3	136.5±17.2	133.3±14.1
Heart rate(bmp)	125±10.7	137±21*	128±15.4

* *p*<0.05, the difference was significant when compared with the value of before modeling establishment.

*SBP* systolic blood pressure; *DBP* diastolic blood pressure

.

### Balloon occlusion combined with surgical hemostasis

The technical success rate of balloon occlusion combined with surgical hemostasis was 100 %. All the arterial lesions in the five dog models were identified accurately and rapidly by angiography, and then were repaired or ligatured successfully. The mean operation time of balloon occlusion was 55 ± 17 min. No significant differences in blood pressure and heart rate were found between different experimental stages mentioned in 5.1 (*p* > 0.05, Table 2)

**Table 2:** Hemodynamics of Balloon occlusion experiment (n=5)

Hemodynamic parameters	Before modeling establish	After modeling establish	Post-operation
SBP(mmHg)	181.6±13.8	186.4±16. 8	184.9±15.2
DBP(mmHg)	135.2±11.9	138.7±16.5	138.3±14.1
Heart rate(bmp)	121±14.3	122±17.7	118±12.4

*SBP* systolic blood pressure; DBP: diastolic blood pressure

.

## Discussion

Injuries to large arteries occur frequently and have become a leading cause of disability and even death after severe disasters, such as destructive earthquake. Timely diagnosis of artery injuries and effective hemostasis are vital for saving lives, and can also improve the prognosis of patients significantly [10–12]. It is also reported that surgical or minimally invasive intervention may be effective in treating vascular injury and hemostasis [7, 13, 14]. Especially, minimally invasive intervention can repair vascular rupture and restore blood flow to the damaged vessels more safely, and has been broadly adopted by clinicians [15, 16]. Analyses of data on the vascular injuries show that minimally invasive technology can lower the misdiagnosis rate, disability rate, and mortality [17]. And angiography and vascular repair should be performed timely on the suspected patients with severe vascular injury as fast as possible. Rasmussen et al. reported that of 488 patients suffering from vascular injury, 28 % (139 patients) received a total of 150 interventional examinations, and the treatment outcomes confirmed the safety and efficacy of the minimally invasive treatment [5]. As a new concept, on-spot vascular intervention can provide effective early treatment for vascular injuries, such as those in disaster areas, where a large number of wounded are presenting with hemorrhage. However, due to a lack of necessary equipment, the emergency minimally invasive interventional treatment can only be performed in the rear hospitals usually far away from the disaster areas. Thus, effective treatments are delayed, and even worse, some people lose their lives in the long distance transport. Therefore, a minimally invasive treatment system with a great mobility and environmental adaptability is needed after a severely destructive disaster. According to the requirements above, the design concept of MIS is to miniaturize the basic equipment of a fixed catheterization lab to facilitate transport and enable on-spot emergency interventional treatment for cardiac and vascular injuries [7]. Meanwhile, it can also function as a standard catheterization laboratory ready to deal with sudden severe cardiovascular injuries or diseases during the long period of post-disaster reconstruction [9].

Aortic injury is often caused by the powerful external force acting on the body. The incidence of aortic injury in daily life has reached 1.5–1.9 % in traumatic injuries [18] and it occurs more frequently in severely destructive disasters due to crash or crush. Rupture of the aorta accounts for a significant proportion of mortality following blunt trauma [19]. Since 80–90 % patients with aortic injury lose their lives on the spot [14, 20], a consensus is reached on strengthening the rapid detection and treatment. In addition, angiography and interventional therapy should be timely performed on patients with suspected aortic injury [21]. The results of our research indicate that MIS can realize on-spot interventional diagnosis and treatment of aortic injuries. However, it should be noted that the uncovered hemostasis and extravasation are the common complications in endovascular stent-graft exclusions, which is mainly due to the stent malapposition or migration [22, 23]. In this research, an extravasation was found after primary endovascular stent-graft exclusions and then a supplementary stent graft was released. As we all know, the evacuation of seriously traumatized patients in severely destructive disasters takes a significantly longer time than in normal conditions, mainly because of the long distance between the disaster areas and rear hospitals, the lack of transportation, as well as the disrupted traffic. Therefore, it is important to achieve complete hemostasis to prevent continuous extravasation, and even hemorrhagic shock or death during the evacuation. Therefore, more attention should be paid to the angiography after endovascular stent-graft exclusions and complete hemostasis, as well as close postoperative monitoring.

In addition to aortic injury, a large number of patients will develop fractures and vascular injuries in limbs shortly after the natural disasters, particularly rapid-onset disasters such as severely destructive earthquake. The main artery injuries of limbs are often caused by direct blunt trauma, crush-related injuries, or stab from the end of fracture. Of these, massive blood loss is often associated with femoral fracture complicated by artery injuries, which leads to serious consequences, such as disability or even death. Normally, once a patient is diagnosed with femoral artery injury, he/she should receive early surgical exploration [24, 25]. The principles of management include controlling hemorrhage effectively and restoring blood supply to the injured limbs as soon as possible to maintain the function. Although the safe time interval from trauma to revascularization is generally considered less than 6–8 h, the evacuation of patients in disaster areas often takes a longer time in severely destructive disasters than under normal conditions. To date, the professional vascular surgery cannot be performed in disaster areas (such as exploration, repair or anastomosis) because of the poor medical conditions in the temporary operation rooms. Amputation is still one of the most common treatments for severe arterial injuries in limbs. In this research, the interventional therapy, such as temporary balloon occlusion, was performed successfully in MIS for treating femoral artery injuries. This treatment has the advantages of detecting the arterial injury spot accurately, less surgical trauma, shorter operative time, and easier bleeding control. With the help of MIS, main arterial injury and hemorrhage can be treated promptly and effectively on the spot by minimally invasive intervention. And for the wounded people, it gains more time for long-distance evacuation and further treatment. Moreover, intervention therapy combined with definitive surgical hemostasis can also be carried out in MIS to further reduce the disability rate and mortality when only a few wounded are on the spot.

There are some limitations in our research. First, the duration from the establishment of the animal model of artery injury to performing interventional treatment is shorter than that in real-life disaster scenes. Second, severe injuries are often accompanied by serious complications in real life, while no complications are studied in this research. Further researches of interventional hemorrhage control techniques, including those combined with surgical operations, should be conducted in the future to improve the diagnosis, treatment and prognosis of more life-threatening injuries in wild and austere environments, such as pelvic fracture complicated by retroperitoneal hemorrhage. In addition, the accurate diagnosis and effective treatment of serious complications of injuries, such as haemorrhagic shock or infection, are critical for saving lives. The diagnosis and treatment of artery injuries with various complications should be carried out in the future by using animal models that simulate severe injuries in real-life disaster scenes.

## Conclusions

In this research, injuries to large arteries are diagnosed accurately by angiography in MIS. In addition, endovascular stent-graft exclusion and balloon occlusion combined with surgical hemostasis are performed successfully. Therefore, it is feasible and effective to perform interventional operations to control hemorrhage of patients with arterial injuries outside the hospital with the help of MIS. The interventional diagnosis and treatment in MIS have the potential to improve the treatment effects of life-threatening injuries in remote and austere environments, such as the severely destructive disasters.

## Abbreviations

MIS: Minimally invasive interventional shelter
DSA: Digital subtraction angiography
SBP: Systolic blood pressure
DBP: Diastolic blood pressure
